# The role of community conversations in facilitating local HIV competence: case study from rural Zimbabwe

**DOI:** 10.1186/1471-2458-13-354

**Published:** 2013-04-17

**Authors:** Catherine Campbell, Mercy Nhamo, Kerry Scott, Claudius Madanhire, Constance Nyamukapa, Morten Skovdal, Simon Gregson

**Affiliations:** 1 Institute of Social Psychology, London School of Economics and Political Science, Houghton Street, WC2A 2AE London, UK; 2 Department of International Health, Johns Hopkins Bloomberg School of Public Health, Baltimore, MD, USA; 3 Biomedical Research and Training Institute, Harare, Zimbabwe; 4 School of Public Health, Imperial College, London, UK; 5 Department of Health Promotion and Development, University of Bergen, Bergen, Norway

**Keywords:** HIV competence, Stigma, Zimbabwe, Community conversations, Community mobilisation

## Abstract

**Background:**

This paper examines the potential for community conversations to strengthen positive responses to HIV in resource-poor environments. Community conversations are an intervention method through which local people work with a facilitator to collectively identify local strengths and challenges and brainstorm potential strategies for solving local problems.

**Methods:**

We conducted 18 community conversations (with six groups at three points in time) with a total of 77 participants in rural Zimbabwe (20% HIV positive). Participants were invited to reflect on how they were responding to the challenges of HIV, both as individuals and in community groups, and to think of ways to better support openness about HIV, kindness towards people living with HIV and greater community uptake of HIV prevention and treatment.

**Results:**

Community conversations contributed to local HIV competence through (1) enabling participants to brainstorm concrete action plans for responding to HIV, (2) providing a forum to develop a sense of common purpose in relation to implementing these, (3) encouraging and challenging participants to overcome fear, denial and passivity, (4) providing an opportunity for participants to move from seeing themselves as passive recipients of information to active problem solvers, and (5) reducing silence and stigma surrounding HIV.

**Conclusions:**

Our discussion cautions that community conversations, while holding great potential to help communities recognize their potential strengths and capacities for responding more effectively to HIV, are not a magic bullet. Poverty, poor harvests and political instability frustrated and limited many participants’ efforts to put their plans into action. On the other hand, support from outside the community, in this case the increasing availability of antiretroviral treatment, played a vital role in enabling communities to challenge stigma and envision new, more positive, ways of responding to the epidemic.

## Background

Whilst community mobilisation is widely advocated as a pillar for an effective HIV response in Africa, much remains to be learned about the mechanisms through which mobilisation exercises its allegedly beneficial effects, and about how best to facilitate such mobilisation. This paper examines the potential of community conversations to promote the development of local HIV competence in resource-poor communities. An HIV competent community is one in which people are able to work together to support appropriate accessing of HIV testing and treatment, the provision of compassionate care for people living with HIV/AIDS (PLWHA), open and non-stigmatizing discussions of HIV, and concrete strategies to prevent new infections [1].

We conducted 18 community conversations with 77 participants led by trained facilitators in rural Zimbabwe. Community members were invited to discuss how they were responding to HIV both as individuals and in community groups, and to come up with strategies to improve local contributions to prevention, care and treatment. In this paper, we discuss ways in which these conversations increased aspects of local HIV competence and the processes through which the approach had its effects. However, we also caution that communities cannot be expected to solve problems through conversations alone; poverty and political turmoil made it difficult for people to put many of their plans into action. Moreover, many positive changes in community attitudes towards HIV (including willingness to talk openly about HIV and go for testing) were closely linked to the increased availability of antiretroviral treatment (ART) at the time of our study, reaffirming that there is no single magic bullet, and that community strengthening approaches such as ours need to be part of a wider toolkit of responses.

### Theoretical framework

#### Community HIV competence

Community involvement is a vital precondition for effective HIV/AIDS management. It plays an important role in enabling health-related behaviours and reducing HIV-transmission [2], and in the reduction of stigma [3]. It is also vital for facilitating timely and appropriate accessing of health and welfare services where these exist [4,5], and for supporting optimal treatment adherence [6,7].

Community involvement is at the core of the concept of HIV competence (also called AIDS competence) developed by Campbell and colleagues to describe the ideal health enabling community environment in the context of HIV/AIDS. They conceptualise HIV competent community contexts as social environments that support and enable people to act in ways that enhance their health and wellbeing [1,8]. An HIV competent community is one where community members work collaboratively to support each another in achieving sexual behaviour change; the reduction of stigma; support for people living with AIDS and their carers; co-operation with volunteers and organisations seeking to provide HIV-prevention and AIDS-care; and effective accessing of existing health services and welfare grants [9,10]

Central to the notion of HIV competence is a group’s ability to engage in critical thinking about local challenges and strengths to develop strategies for improving care of PLWHA, prevention of new infections and appropriate accessing of available testing and treatment services. Education theorist and social activist Paulo Freire argues that critical thinking by marginalized groups can often constitute a key precondition for action and health-enhancing individual and social change [11]. Freire’s ‘transformative communication’ approach maps out the processes through which subjects pose problems and critically examine everyday life experiences through discussion [11-13]. In this paper we take up Freire’s theory of social change by seeking to implement an intervention that engages communities coping with high HIV rates in critical thinking and the development of action plans as a precursor to change [14].

Community dialogue can enable people to translate and digest new information in order to apply it effectively to their lives. Such discussions may form the starting point from which people begin to take action to tackle the impacts of the unequal social relations that often underlie ill-health [15]. Fraser [16] argues that, in unequal societies, marginalised groups (in our case, impoverished rural Zimbabweans) are excluded from the public sphere in which governments and other leaders make significant decisions about the shape of social life. They tend to lack the confidence, skills and social legitimacy to advance their needs and interests. She suggests the creation of ‘counter-public’ spheres, which are safe separate spaces in which marginalised groups can retreat, to develop and ‘rehearse’ the types of critical arguments they will eventually take into the dominant public sphere Based on the assumption that epidemics arise when existing dominant ways of understanding and responding to problems are ineffective [17], community conversations adopt Fraser’s approach by attempting to create alternative spaces that enable people to discuss issues away from mainstream social environments that promote the status quo, thereby opening space for new ways of thinking and questioning.

#### Community conversations

The term ‘community conversation’ describes discussions among local people, guided by a trained facilitator, that support critical thinking and problem solving around key community issues [18]. Community conversations have been used to address a range of issues including: mental health stigma among ethnic minorities in Scotland [19,20] increasing employment opportunities for high school youth with disabilities [21]; improving early childhood educational alignment [22]; efforts to determine health issues and better meet health needs among populations such as rural people, particularly Native Americans, in North Dakota [23] and elderly Cambodian refugees in Massachusetts [24]. The term has been applied to post-performance or talkback sessions in the field of community theatre or film screenings on issues ranging from the incarceration of African American men to environmental justice [18,25].

Whilst the approach is widely used, there is little or no systematic account of the methodology of community conversations in the peer-reviewed academic literature. Moreover, most of the few articles that exist discuss initiatives in North America, with almost no academic writing on the use of community conversations to address issues in resource-poor countries. However, in the grey literature, the United Nations Development Program (UNDP) has pioneered the use of this method in resource-poor communities in Africa, particularly surrounding gender and HIV issues in Ethiopia [26]. In this context, the effectiveness of this methodology in changing community attitudes and practices first came to light through the work of Ethiopian women’s rights campaigner Dr. Bogaletch Gebre. She founded the African women’s self-help centre Kembatti Mentti Gezzimma (KMG) and has been credited with reducing the practice of female genital mutilation in Ethiopia [27] primarily through developing and implementing the community conversations technique.

Although some researchers loosely use the term ‘community conversation’ to describe an informal focus group [28] or discursive trends in a community (i.e. the ways people talk about issues in their lives) [29], community conversations are generally considered to be a unique and new intervention type that is distinct from focus groups in several key ways [25]. First, community conversations are focused on generating action plans. They have an explicit ‘problem solving’ agenda, aiming to spur critical thought that enables people to formulate local solutions to local issues. By comparison, focus groups are more research oriented, aiming to gather information about social relations and understandings. Second, community conversations explicitly aim to change participants’ worldviews and conceptions of what is possible, while focus groups aim to understand things as they are.

Aspects of the way in which we used the community conversations approach resonate with Participatory Action Research (PAR) [30] insofar as community conversations are interventions which seek to change some aspect of the social world at the same time as conducting research into this transformation process. However, our approach differed from PAR, in that PAR generally involves research participants in decisions about the formulation of research questions, and/or in the analysis and interpretation of research findings. This was not the case in our study, where community participants were only involved in the social change exercise, but not in any aspect of research design or analysis.

#### Conducting community conversations

Community conversations are used in the context of solving social problems. They involve posing questions and thinking points about why problematic social situations are the way they are, what actual and latent local responses and strengths exist in the community to tackle these, and how problematic social relations could be improved. The community conversations literature often specifies that facilitators should be local people who are invested in the research community [26] and known and trusted by conversation participants [27]. Such a facilitator brings together a group of people who consider themselves to be members of the same community, where community is understood as a group of people living in the same area and experiencing similar local strengths and challenges.

In our study, the facilitators of our community conversations of interest (Nhamo and Madanhire, both co-authors of this paper) were both community insiders and outsiders in different ways. Both shared the ethnicity, home language and province of origin of the research participants. They were black Shona-speaking Zimbabweans who had grown up in the province in which the research was conducted – albeit some distance from the research site. However in terms of social class, educational status and current residential location, the facilitators were worlds away from the informants. They were both post-graduate educated, middle class professional researchers, one resident in the UK and the other employed by a prestigious research institute in Zimbabwe’s capital city some distance away. In this respect they were definitely community outsiders, and as will be discussed below, this was how the research participants regarded them. The potential implications of the facilitators’ social positioning are discussed in detail later in the paper.

On the basis of research team experience of conducting similar group-based research and intervention approaches in South Africa, Kenya and Zimbabwe, six to ten people were chosen as the optimal number of community conversation participants in order to have groups small enough to ensure that all members had an opportunity to speak, but large enough to maximize discussion and diversity of opinion.

The facilitator poses questions to the group and invites discussion, emphasizing the importance of respectful disagreement, honesty and problem solving [18]. Dr. Gebre, the pioneer of community conversations in Ethiopia, recognizes that conversations may have to take up practical and immediately pressing issues in a community (such as a broken bridge) before moving on to deeper, systemic or taboo issues (such as female genital mutilation or HIV) [27]. The facilitator does not attempt to teach or advise community members; rather, his or her role is to bring out pre-existing community understandings and strengths and to encourage the community to analyse and solve local problems. As problems and solutions are discussed, the facilitator guides the participants to develop concrete action plans [18]. Ideally, the facilitator will convene additional conversations on an on-going basis to discuss how implementing the action plans is going and develop additional strategies to overcome challenges.

## Methods

### Context: HIV in Zimbabwe

The study was conducted in an area of rural Zimbabwe with an HIV prevalence rate of approximately 20% [31]. Residents of the region are primarily subsistence farmers or workers in mining enterprises, large commercial forestry operations, or tea estates. Most live in rural homesteads (compounds with several mud and thatch houses, a pit-latrine and animal pens), often without electricity. Many families have absent members working in cities, mines and commercial agricultural estates, some of whom send money back to the rural areas. Poverty is a major challenge and many local people receive food aid from the government and international organizations.

The 2000s, as a whole, was a period of economic and political instability in Zimbabwe. The data for current study were collected between the period leading up to the presidential election run-off in June 2008 and the establishment of the Government of National Unity in February 2009. This period was marked by intense political strife and hyperinflation. The inflation rate had reached 1281% by 2006 and increased subsequently to 66,212% in 2007 and 231,150,888% in 2008. By October 2008, the exchange rate had fallen to 2,621,984,228 Zimbabwe dollars to the US$ and the Zimbabwe dollar was replaced with a multi-currency system early in 2009.

Anti-retroviral therapy (ART) for HIV became significantly more widely available across Zimbabwe in the late 2000s, as a result of concerted government and partner aid organization efforts. By December 2009, 218,589 people in Zimbabwe, about half of those in need, were on free ART through the public health sector [32]. In our area of interest, free antiretroviral drugs became increasingly available in hospitals from late 2008. However, significant barriers to access remained, including high costs of transportation to and from hospitals, accessing a doctor (of which there were very few) to initiate treatment, and paying the hospital fee (usually $1US) [6] as well as for men to overcome their resistance to being associated with HIV [33,34].

### The data set

Eighteen community conversations were conducted in batches of six over the course of three rounds: May 2008, September 2008, and February 2009. This time period enabled an exploration of changing attitudes over time, particularly as ART became increasingly accessible from 2008 to 2009. Both study locations had been sites for earlier HIV/AIDS-related research and intervention, and our conversation facilitators were already known to local village guides. Village guides introduced our facilitators to leaders of six local faith-based community organisations. In meetings with these local grassroots leaders, our facilitators explained that the research aimed to use community groups to identify ways in which local people might respond more effectively to HIV/AIDS. They explained that the groups sought to develop locally feasible plans that could draw on existing community resources – rather than relying on funding or expertise from outside NGOs or the government as had often been the case in health and social development interventions in the past. Leaders were enthusiastic about assisting in recruitment, citing two reasons: the severity of the impact of HIV/AIDS on local people, and the recent relative dearth of outside support for responding in conditions of government resource constraints and low levels of NGO activity due to current economic and political instability. The leaders themselves recruited informants from their own networks, approaching people they knew personally and inviting them to participate. We have no way of knowing how representative participants were of the general village community, and have no record of the refusal rate of those they asked to participate. In the face of high levels of stigma, no specific effort was made to single out people living with HIV/AIDS for inclusion, or to identify such people in the group settings. However, given that 20% of local people were HIV positive at the time of this study, we can cautiously assume that some would have been included amongst our informants.

We hoped to have the same participants in each of the six groups at rounds one, two and three in order to follow up and ask them about changes they had observed between conversations. However, because the economic and political situation limited travel at times, many groups during the second and third rounds included new participants to fill empty spaces. Overall, 77 different people participated--35 more than would have if the same participants attended all three rounds. The second round, in September 2008, was during a particularly fraught political period and, as such, we faced the most difficulties accessing repeat attendees. In the second round, half of most groups were new participants with one group being an entirely new group of people. The conversation groups in round three, in February 2009, included a far higher percentage of repeat participants. See Table 1 for details.

**Table 1:** Description of dataset

**Time**	**Location**	**Participants**	**Group code**
T1 (May 2008)	Beacon Hill	7 (4 male, 3 female)	NA
	Beacon Hill	7 (4 male, 3 female)	NB
	Beacon Hill	6 (2 male, 4 female)	NC
	St Magdalena’s	7 (3 male, 4 female)	StA
	St Magdalena’s	9 (5 male, 4 female)	StB
	St Magdalena’s	6 (4 male, 2 female)	StC
T2 (Sept 2008)	Beacon Hill	6 (2 male, 4 female) [2 old, 4 new]	NA
	Beacon Hill	6 (4 male, 2 female) [4 old, 2 new]	NB
	Beacon Hill	8 (3 male, 5 female) [0 old, 8 new]	NC
	St Magdalena’s	7 (2 male, 5 female) [3 old, 4 new]	StA
	St Magdalena’s	8 (3 male, 5 female) [4 old, 4 new]	StB
	St Magdalena’s	8 (4 male, 4 female) [4 old, 4 new]	StC
T3 (Feb 2009)	Beacon Hill	7 (3 male, 4 female) [7 old, 0 new]	NA
	Beacon Hill	6 (4 male, 2 female) [6 old, 0 new]	NB
	Beacon Hill	7 (3 male, 4 female) [6 old, 1 new]	NC
	St Magdalena’s	9 (4 male, 5 female) [7 old, 2 new]	StA
	St Magdalena’s	9 (3 male, 6 female) [8 old, 1 new]	StB
	St Magdalena’s	8 (4 male, 4 female) [3 old, 5 new]	StC
Total		77 individuals	

We conducted the conversations at two locations, which are identified with pseudonyms to respect participant confidentiality (three groups in Beacon Hill and three in St Magdalena’s). Beacon Hill is a small township with a high rate of migration and informal work (particularly small scale sale of goods at the roadside and unregulated diamond panning), meaning fewer long term relationships developed between residents. In contrast, St Magdalena’s is a rural area with strong, deeply rooted community relationships, centred on a mission station and hospital. Most residents farm their own land.

As indicated above, community conversations were conducted by two trained Zimbabwean facilitators (the second and fourth authors), one female and one male. Both spoke Shona, the local language, as their mother tongue.

All participants were given a large block of soap as a token of gratitude for their time. Ethical approval for the study was granted by the Medical Research Council of Zimbabwe, the Imperial College Research Ethics Committee, United Kingdom, and Social Psychology Research Ethics Committee at the London School of Economics. Consent forms were used, outlining principles of anonymity, confidentiality and freedom to withdraw from participation at any stage. Informants either signed, or used a thumbprint, to indicate their consent.

### Procedure, coding and analysis

The facilitators guided the conversations using topic guides designed to elicit information about HIV-related problems and potential strategies for local responses. Topics included: who PLWHA disclosed their HIV status to; whether HIV stigma was a problem in the community; where, when and how people discussed HIV avoidance, testing and treatment; the physical and financial accessibility and social acceptability of HIV testing and treatment services; what local efforts were being made to support PLWHA and reduce new infections; problems encountered when attempting to help PLWHA or talk about HIV; and ideas to reduce risky behaviour, stigmatizing attitudes and poor quality care for PLWHA. Each conversation also featured two ‘break out sessions’ where the group was split into two. The resulting smaller groups were asked to list ideas of how they could reduce stigma in the community (which previous research and the research team’s informal experience had identified as a key obstacle to effective HIV responses [35]) and what efforts they were already making to support PLWHA and encourage prevention, testing and treatment. The groups came back together and presented and discussed their ideas.

The community conversations were generally animated and ranged in length from one to two hours, with most being approximately one and a half hours. Women and men participated equally. Groups were conducted in Shona and audio-taped. After translation to English and transcription, the transcripts were read and re-read before coding. Using NVivo qualitative analysis software, we coded text segments relevant to our research interest in how community conversations can impact local HIV competence. We thus coded the following: participant comments and reflections on the community conversations and facilitators (what they liked or did not like, thanking facilitators); discussions of strategies to respond to HIV developed during the community conversations; comments on HIV-related issues in the community getting better or worse; comments on community member support for one another or cruelty towards one another; and comments on gaining HIV knowledge and gaining intent to respond differently to HIV. This process generated a total of 23 basic themes, using Attride-Stirling’s [36] method of interpretative thematic analysis, a data sorting and clustering process which identifies descriptive *basic themes* (text segments), which are then grouped into increasingly abstract higher order *organising themes* and highest order *global themes*. These 23 basic themes were progressively grouped and regrouped in ways that identified five pathways through which the community conversations may have contributed to the development of HIV competence, with each pathway constituting an organising theme. Another two organising themes reflected information about factors that may have facilitated or hindered the success of the community conversations in generating HIV competence. These 23 basic, 7 organising and 2 global themes are presented in Table 2. The organising themes– make up the sub-headings in our ‘Results and Discussion’ section, below.

**Table 2:** Coding framework

**Basic themes**	**Issues discussed in CCs**	**Organising themes**	**Global themes**
Condom distribution	• Improving HIV services	(1) CCs allow community members to develop concrete action plans to cope with HIV	Community conversations (CCs) facilitate HIV competence
	• How best to care for PLWHA		
Distributing food			
	Strategies to reduce stigma		(Part I)
Keeping vegetable gardens			
Home based care			
Engaging with the Church			
Participants felt motivated	• Participants want to play a role in the HIV response	(2) CCs provide community members with an opportunity to work with outside facilitators	
	• Careful and respectful facilitation by outsiders		
encouraged to action their plans			
	• Facilitators enabled new ways of thinking		
Valued by facilitators			
Challenging damaging norms			
Local strengths	• Recognition of the importance of a common purpose	(3) CCs allow community members to work towards a common goal	
Local barriers to action			
	• Importance of taboo subjects to be discussed and ways to collectively overcome stigma		
Collective action for more openness			
Recognition of lack of individual agency	• Need to act, develop solutions and translation information into action	(4) CCs can facilitate problem solving	
Potential of the collective to turn information into action			
Sharing of personal stories	• Recognition that HIV is not a family issue but a community responsibility	(5) CCs can overcome HIV-related silence and stigma	
Recognising the scale of HIV			
	• Improvements in HIV communication		
Easier to talk about HIV			
Good health because of ART	• ART has enabled local efforts to implement action plans	(6) Facilitators of HIV competence	Contextual factors influencing HIV competence
ART has meant HIV is no longer a death sentence			
			(Part II)
Poverty	• Poverty, droughts and inflation made it sometimes difficult for community members to respond to HIV as they wanted.	(7) Barriers to HIV competence	
Poor harvests			
Risky behaviours	• Poverty and hunger fuelled risky sexual behaviour		
	• Political situation meant some community members feared meeting in groups		
Political upheaval			

## Results and discussion

### Overview

What role did community conversations play in increasing HIV competence amongst participants? The data suggest that, while community conversations are not a magic bullet, they potentially contribute to developing HIV competence through: (1) enabling participants to develop concrete and practical action plans to combat stigma and better support PLWHA; (2) challenging participants to think creatively and take positive action with the encouragement of facilitators; (3) working towards a common goal and being able to discuss taboo subjects; (4) encouraging participants to move from seeing themselves as passive recipients of HIV-related information to active problem solvers, and (5) providing an opportunity for participants to conceive of ways to move from information to action.

Each of these points is discussed in detail in turn in Part I below. Part II discusses the contextual factors beyond the control of community members that (6) facilitated and (7) hindered the capacity of community conversations to build HIV competence. On the one hand, the increasing availability of ART from late 2008 was a particularly supportive context for the goals of the intervention. On the other hand, negative contextual features, particularly severe poverty, poor harvests and political upheaval, limited the feasibility of action plans.

The conversations frequently generated debate and storytelling among participants, with some people recounting highly emotional—sometimes tragic and sometimes inspiring—personal stories of coping with HIV/AIDS among their family or friends. Community members came up with many concrete action plans and reported some success at enacting these plans during follow-up conversations. In addition, there was evidence of critical discussion with participants debating whether or not HIV stigma existed and if it could be reduced, the roles of men and women and young and old people in the spread of HIV, and the practicality of various ideas of better supporting PLWHA and reducing new infections, discussed further below. Participants frequently told the facilitators that participation in the conversations was improving their capacity to respond more positively to the challenges of HIV by breaking the silence around stigma and encouraging discussion. There were countless instances, particularly in the third round of conversations, where participants described a great deal of change in the community’s attitude towards HIV, with many suggesting that the community conversations had played a role in this.

Before detailing our specific findings from these community conversations we must emphasize that there are many caveats about the limitations of reported attitude or behaviour change in the HIV/AIDS field. Participant reports cannot be taken as conclusive evidence that changes occurred in the broader communities outside the conversations, or that any changes can be attributed to the conversations. Participants may have felt an incentive to please the facilitators by emphasizing the effectiveness of the intervention, perhaps in hopes of maintaining links to the facilitators or accessing future assistance. Individuals may have sought to exaggerate their virtues in order to impress other participants. Recording the conversations may also have inspired people to present themselves in a particularly positive light. In addition to features of the within-group dynamics, group participants themselves referred to countless other environmental factors, outside of the group contexts, that enable or frustrate community efforts to cope effectively with HIV, discussed in part II. In the local context, a particularly significant development between rounds one and three of our groups was the increase in ART availability in the region through the efforts of the Zimbabwe health ministry and foreign donors. Such a significant and positive external event would have strongly reinforced group efforts to promote positive and creative dialogue and HIV-related action plans.

In this paper, rather than focusing on whether community conversations changed behaviour or attitudes in the community outside of the conversations, we are concerned with examining the extent to which community conversations were able to function as social spaces for critical thinking and the development of action plans amongst participants, which Freire would argue were a necessary precondition for community level change. However, as emphasised above, we cannot claim that these are a sufficient condition for behaviour change. We acknowledge that a range of other factors – from situational factors to individual differences amongst participants – would mediate the translation of plans into actions that might result in positive health outcomes.

### Part I: In what ways did community conversations contribute to HIV competence?

We now present our detailed findings on the five specific elements of community conversations that appeared to facilitate the building of HIV competence in our study.

#### (1) Conversations enabled participants to develop concrete, practical action plans to better cope with HIV

An HIV competent community is one in which members conceive of concrete ways in which they can contribute to better supporting PLWHA, reducing stigma and new infections, and encourage access to available HIV testing and treatment services. Our findings suggest that the community conversations (CCs) were effective in supporting participants to jointly come up with possible new strategies to cope with HIV: participants brainstormed how better to care for PLWHA, how to reduce HIV stigma and how to encourage prevention, testing and treatment. For example, some participants said they had decided to distribute condoms and to teach people “that AIDS is not a curse from God, but just a disease” (JO, male, Time 3, StA). In other instances, participants collaborated to develop more effective means of helping PLWHA. In the following, SY offers food assistance and his offer is taken up by MA:

**SY (male):** I want to say that I might be out of touch on some of these things because I am actually busy with work at my plot most of the time. But I want to ask anyone here to let me know if they find any challenges with regards to food for any of the patients^a^ they visit. I am more than willing to assist with food. They can tell me, I have maize which I think can assist others in need. So next time when you visit let me know what challenges you have where I may assist, I don’t mean to say I will give everything you need but I will definitely do something about it.

**MA (female):** I also want to thank [SY] for offering to help, I also have a certain couple who are HIV positive and … their worry is food. So [SY] I will definitely approach you after this session for those people. (Time 3, StA)

Participants also developed concrete action plans such as taking turns in maintaining vegetable gardens for food to donate to PLWHA and approaching church leaders to encourage additional discussions about HIV:

This has been a very hard year and we really had to struggle because sometimes these patients expect to receive some material assistance over and above our prayers and counselling and keeping them company. … That’s why we came up with an idea of gardens so that we can supply them with vegetables whenever we visit them. (DO, female, Time 3, StA)

…In our church I have approached my pastor and the bishop who came here after you guys [the group facilitators] left and I put the issue of AIDS forward to them. They received my message very well and began to encourage people in the church to set up a fund that is meant to benefit HIV/AIDS sufferers. (EU, female, Time 3, StB)

Other concrete strategies to help reduce stigma and help PLWHA included: helping bathe and cook for the children of PLWHA, donating fresh milk and firewood as well as vegetables to families with HIV-positive members, praying for PLWHA (a simple but significant way of showing kindness), ploughing, planting and harvesting the fields of people too sick to do so themselves and maintaining normal community relationships with HIV-positive people (such as continuing to visit their homes and ensuring they are able to keep their positions in the church). For example, ME presented her simple but profound idea on how to approach PLWHA in non-stigmatizing ways:

**ME:**I think we should at least try to be free to these patients and get them to talk, to be friendly and avoid viewing them as helpless patients, which happens when we show a lot of pity for them. If we were friends we should see them as our friend and try to talk them as if nothing has changed about them. (female, Time 3, NB)

Many participants had been helping care for PLWHA within their home for years before the community conversations began, but said that they had previously felt constrained in talking openly about their experiences given the very high levels of HIV stigma. Through having the opportunity to speak about the needs of PLWHA and developing strategies to address these needs with community members beyond their immediate family, participants were able to reframe HIV from a family-level issue to a community-level issue.

Whilst participants proposed some strategies to reduce the spread of HIV and promote testing and treatment, these ideas were often not as concrete or practical as their strategies to better support PLWHA. Many planned to verbally encourage people they knew who to suffered repeated illnesses to go for testing and warn young people against pre-marital sex. A few people reported having tried to convince sex workers to stop selling sex. These strategies were vague, taking little account of the underlying social and economic drivers of risky sexual behaviour. The strategy of condemning the risky behaviour of others is often used to distance those who condemn from a sense of their own personal vulnerability (i.e. focusing on young people or sex workers as those at greatest risk of HIV, rather than acknowledging how people ‘like them’ were also at risk of infection).

Nonetheless, participants did share some practical solutions on the subject of prevention, testing and treatment. Most commonly these included helping people get to the clinic through donating money for transportation or helping to physically carry them if they were very ill; getting community leaders (village chiefs and church leaders) to talk more about HIV in forums such as funerals and Sunday services; and strategically accessing external support (mainly NGO help) for the community. This latter idea was mostly exhibited through participants asking the facilitators to run the same intervention in additional areas, such as schools and churches. For example, one participant said: “…you guys can make a difference if you take this [CC] programme to schools” (AI, female, Time 2, StC) and another said “I think they [young people] also need to be targeted with programmes like this one because when we try to warn them they would just brush aside everything we say as just rhetoric. They don’t value what we say” (OT, female, Time 1, NB). Participants recognized the facilitators as a link to resources and knew that their symbolic status added salience and credibility to HIV messages and thus appealed to them to help the community.

Developing concrete ideas regarding how to help the community better support PLWHA and encourage prevention, testing and treatment was a positive process for two reasons: first and most obvious, having an action plan increases the likelihood of implementing positive changes because participants have concrete ideas of what they can do. There were many reports by participants in the second and third conversation rounds suggesting that they did in fact take up these action plans. Second, by encouraging the development of action plans, participants began to frame HIV as something they could positively influence.

#### (2) Participants were encouraged and challenged by involvement of outside facilitators

Formal accounts of the community conversations approach emphasise the use of a trained local facilitator (Shetty, 2007). In this regard we diverged somewhat from the formal guidelines. While both our facilitators were Zimbabwean and spoke the Shona language of the participants, neither of them had personal links with our two study communities. Furthermore, both of them were university graduates and employed in professional research jobs whereas the CC participants were less formally educated and farmers or manual labourers. Our findings suggest that a key driver of community conversation success was participants view of the facilitators as high status community outsiders. Community members appeared to trust and relate to them but also expressed respect for them and gratitude that they had come to the region and cared to help. Their presence seemed to appeal to participants and strengthen the effectiveness of the conversations for three main reasons, discussed below.

##### Participants felt motivated by facilitators and inspired to act

Respondents repeatedly said that the involvement of the facilitators in the CCs inspired them to put their plans into action. They said that they were keen not to ‘let down’ the facilitators, given the trouble they were taking to implement the intervention.

**TH:** We talked about HIV at community gatherings and gave soap to PLWHA so that everyone in the community can see what you have been teaching us. Since you came here there is now a big difference. So we wanted others to know that there is this programme. (female, Time 3, StB)

Participants seemed to take the expectation that they implement their action plans very seriously:

**AN:** I visited someone with HIV and cleaned her home because we had been taught by you that we should help those who are sick, so I did it so that I can put what I have learnt in practice, and I also wanted others to know how they can treat their patients. (female)

**PH:** I also helped because you taught us to do that. (female, Time 3, StB)

It is noteworthy that participants mentioned having been ‘taught’ when CC facilitators specifically avoided imparting any HIV related messages or suggesting strategies. This could indicate that local people were keen to give credit to the facilitators for gains made. It could also indicate that participants perceived having been taught when in fact the CCs had drawn out latent understandings and conceptualizations already present among the group.

##### Participants felt valued, not forgotten

At the time when the CCs were held, many foreign NGOs had ceased operations in Zimbabwe due to the unstable political and economic climate, and opportunities for community group meetings and activities were restricted by political conflict and laws requiring police permission for public gatherings. Against this background, participants expressed a sense of ‘honour’ at having had the opportunity to participate in the groups, and an associated sense of responsibility to try to generate some positive community gains from their involvement. Participants expressed a sense of having been abandoned by other organizations, as the following quotations illustrate:

**NB:** They [an NGO] used to give but not on monthly basis, but they have just vanished (SE, female, Time 2)

**NC:** They [an NGO] stopped some few weeks before the March elections and they have not resumed their activities since then (SI, male, Time 1)

The ‘participation fatigue’ that is often cited as undermining peoples motivation to engage in AIDS programmes in other contexts (where participants tire of engaging in an on-going community intervention, due to a growing sense that the effort of attending outweighs the benefits) [37] did not appear to be an issue. In contrast, participants were very eager to engage with the community conversations and were thankful and heartened to see outsiders come to help.

**MA:** I just want to thank you guys for coming here, it shows a lot of commitment on your part, and I hope this is not the last time we are seeing you here and we hope to move together as we fight stigma. We hope soon you will be able to come to our church and give a talk as I requested. I hope you will consider that request. Some organisations who used to work here have completely forgotten us because since the days when they were stopped by the political situation we never saw them back, we just hope they are considering coming back again. (male, Time 3, StC)

With so few resources coming from the outside to assist their communities, the presence of these facilitators appeared to represent a valuable link to external support and evidence that poor rural people had not been forgotten.

##### Facilitators challenged participants to think in new ways about HIV issues that were locally seen as normal or unchangeable

The facilitators performed another role that appeared to enable these conversations to spur critical thinking and the development of feasible action plans: they challenged normative worldviews and limiting behavioural repertoires. In this respect it appears to have been vital that outsiders injected new ideas into the CC dialogues. As the following quotations illustrate, the facilitators took care not to impose new ideas in a prescriptive way, rather seeking to feed them into the discussions to serve as the raw materials for the development of new ways of being and seeing.

The following dialogue (Time 1, NC) shows the facilitator asking critical questions of participants to encourage them to move away from a simplistic understanding of an HIV-related issue---in this case young people’s promiscuity and associated behavioural problems, such as acting ‘spoiled’ (i.e. not helping at home and desiring consumer goods), acting ‘sassy’ (i.e. not adhering to parental instruction) and skipping church. One participant, with the agreement of the larger group, presented the opinion that the government was contributing to their children’s bad behaviour by enacting a law that recognised people over 18 as adults, and therefore beyond the legal control of their families. Blaming this law enables community members to avoid discussing local issues leading to youth promiscuity, and fails to admit that ‘youth promiscuity’ concerns children much younger than 18 years. The facilitator asks questions to get participants to think through their understanding of the issue:

**Facilitator**: You are the ones who are facing all these challenges; what do you think should be done?

**NI, male**: … I think the government has also a role to play. I think the government is enacting some laws that make it hard for us to control our kids.

**Facilitator**: Which laws are these?

**NI**: The government says that at 18 years the child is now free to do whatever they want… [This law is on the legal age of majority for voting] That’s when our children begin to tell us that they are adults and no longer want parental guidance. Ladies, am I not telling the truth here?

[Some noises suggest agreement]

**Facilitator**: Do you mean your youths are only giving you problems when they are 18. And before that they would have been well behaved all along?

**NI**: They begin [misbehaving] at around 14.

**Facilitator**: So would the same law cover them?

**NI**: No.

The facilitator did not propose any alternate understanding and did not teach or impose his views. Instead, he gently pushed participants to see the issue in a new way. After the above exchange, the participants came up with other ideas (rather than blaming the law recognizing adulthood at age 18) to address the risky sexual behaviour of young people. Ideas included adding more Christian education to the schools and encouraging parents to be stricter with their children. While these ideas are not necessarily revolutionary, they are better than blaming an unrelated government law and they show evidence of participants thinking of community action plans to reduce risky sexual behaviour among young people.

Participants emphasized how deeply changed they were by taking part in the community conversations and linked the experience closely to the facilitators. For instance, one participant said: “You gave us the impetus to do this, you made us do this and we can't stop it now” (AN. female, Time 3, StC). As mentioned earlier, we must consider the chance that participants may have been overemphasizing the impact of the intervention in order to please the facilitators, perhaps in hopes of ensuring future visits and programming. Nonetheless, as KU, female, below, suggests, being questioned by outsiders often forces new ways of thinking and seeing the world, something participants valued and wished others could experience:

**KU:** I encourage you to even come to our church and talk to people the way you were talking to us--by asking some questions we learn a lot and one would wish that everyone could get this opportunity. (female, Time 3, StC)

Participants frequently asked the facilitators to run the same intervention with additional groups. These requests suggest that participants valued the community conversations and believed others in the community would also benefit from participating.

#### (3) CCs constituted a forum in which people could develop sense of community, common purpose

Bringing community members together and encouraging them to discuss their local strengths and challenges appeared able to bolster a sense of common purpose. This was particularly evident in Beacon Hill, a community of more transient traders and informal labours, without the same level of entrenched family and neighbourhood ties as the more agrarian St Magdalena’s.

**TA:** Most of the people in this community are just resident here; they have relatives far away, so they also feel loved when we help them, people become more united and feel more related than they are. I am sure your coming here has helped us to begin to feel like we are just all related. I think you have helped us to bring us together and begin to see other people in this community as family even though we are not related. (female, Time 3, NB)

**TE:** After you left us last time we sat down as a group and decided that we should work together and coordinate our efforts, so we agreed that we meet regularly and talk about the patients that we would be having in this community from time to time so that we find ways of helping them where we can. Many people in this community come from other areas – and though we are strangers we decided that the only source of our help is each other. So we decided that we should visit HIV/AIDS sufferers and bring them what we can afford, sometimes we go to see the patient and ask them what they want to eat, then we try to make their desired things available. (female, Time 3, NB)

Participants also commented on the conversations’ role in unifying church groups and helping HIV-positive people become more open about disclosing their status:

**TH:** … Since you came here we have been holding inter-church gatherings to make sure everyone is on the same footing. These groups have brought more unity among churches, and it has recently been said all churches should also talk about HIV/AIDS during their services. Now it seems HIV/AIDS sufferers are now feeling proud. Now that you have come here they will say “we have HIV” because once they say that people begin to be very helpful. (female, Time 3, NB)

While community conversations need to take place among people who already consider themselves to be united as a community, our study suggests they can facilitate a deeper sense of collaboration and common purpose amongst participants.

#### (4) CCs encouraged participants to move from passive recipients of HIV-related information to active problem solvers

Participants credited the conversations with helping them envision themselves as agents who could contribute to building local HIV competence. Many mentioned that local knowledge of HIV was sound and there was no need for more information. Instead, they expressed a lack of collective agency to move from information to action.

**SI:** Your coming here is helping us with a lot of things. Though we knew about HIV/AIDS we really never thought we could also do something ourselves until you came and talked to us. I personally was at least doing my little part but I never thought we could actually work as a group and achieve something. Now I find that when we go as a group we lighten the burden very much for the care giver. The caregiver is normally used to only seeing one visitor after a while. When we visit, the women start washing and cleaning the house, while the men will help to lift the patient, changing their position (male, Time 3, StA)

KU below again emphasizes that the conversations not only taught him about HIV but also made him think critically about his own capacity to do something:

**KU:** I have learnt a lot from attending your sessions. I have learnt that I should do something to assist HIV/AIDS sufferers in our community. After I took it to our church we began visiting HIV/AIDS sufferers regularly - bringing whatever small things we can, be it a piece of soap or just some bananas, and continuing to visit and pray for them. Some patients had food but needed assistance to stand up or be carried to the toilet, so I and my group assisted some people in that way. Sometimes we just talked to the patients, or helped them fetch some firewood. We were trying to give them hope. (female, Time 3, StA)

JA’s statement, below, gets to the heart of the value of community conversations:

Action speaks louder than words. People are now saturated with information so I will try to show what I mean by being extra good to patients. (JA, male, Time 3, NC)

At this stage in the epidemic, people are ‘saturated’ with information about HIV. For JA, the CCs offered the possibility of turning such information into action.

#### (5) Community conversations reduced the silence and stigma surrounding HIV

By bringing people together and encouraging open discussion of HIV, community conversations reduced the silence surrounding HIV. Participants shared personal stories about HIV and came to see that almost every family was somehow affected by the disease. In the following, CL comments on how prior to the programme, supporting PLWHA were seen as a private family issue rather than a community responsibility:

[The community conversations] have helped us to be more serious on taking care of the patients. It has helped us to realize that they are people just like us, they need us, they need our love. Because long back we use to think that the only patient we should help is someone who is within my own household, but your coming helped me to realize that I should take care of everybody, I should help. (CL, Time 3, NC)

Bringing people together to talk about HIV with facilitators who ask challenging questions about the status quo and encourage new ways of thinking can break the silence and reduce stigma. Ethel (below) reflects that the discussions have made it easier to talk about HIV:

…Now we know how we can work together. We are no longer shy or afraid to talk about HIV/AIDS. You have helped so much and now we can discuss and talk to each other freely and now we are able to continue meeting as a group even after you guys have gone. You guys have made us mature and this had moulded us by getting knowledge and skills from sharing with others. (ET, female, Time 3, StB)

The conversations appear to have forced discussion about HIV and enabled participants to reframe their understandings and attitudes towards PLWHA. WI (male, Time 3, StA) reports that the conversations have “been very effective in changing how we perceive HIV sufferers.”

### Part II: Contextual influences that facilitated or hindered effectiveness of the community conversations

HIV competence of a community is influenced by a complex array of factors that lie beyond the reach of a single specific intervention. A large research literature highlights contextual drivers of community responses to HIV, such as systemic poverty and entrenched gender norms. We now turn to examine contextual factors that (1) facilitated or (2) hindered participants’ efforts to implement their action plans.

#### (6) Facilitators: ART availability

In the third round of CCs, participants repeatedly referred to the role of ART availability in bolstering their efforts to implement the action plans formulated in the CCs. Participants closely linked a perceived reduction in HIV stigma to the availability of treatment. For example, in the following quotation, AM links improved levels of testing and reduced stigma to opportunities to access ARVs:

**AM:** There was a person we didn’t think would survive into the next month, but we encouraged them to consider going for HIV tests and to see the doctor. Now that person is looking very healthy. We have also asked the doctor to make regular visits so that St Magdalena’s people no longer need to go to R to collect their monthly prescriptions or for medical check-ups. So I think what you have done here is working very well, since you have been here we go about our villages talking about what we have learned and encouraging the sick to get tested so they can be put on ARVs. (female, Time 3, NC)

AM attributes this change to the CCs; however, if it weren’t for the newly available treatment options, the idea of encouraging people to go for testing would not be as compelling. The knowledge that treatment is available reduces the stress and trauma of finding out one’s status, opening up the possibility for HIV-positive people to return to health, reducing the burden on families and communities. DO, in the following quotation, reinforces the link between improved community attitudes towards HIV and treatment availability:

**DO:** Now that we have the information and the facility where people can access ARVs at St Magdalena’s, I think people are beginning to realize that HIV/AIDS is not a death sentence (female, Time 3, StA)

#### (7) Hinderers: Poverty, poor harvests, and political upheaval

As stated above, the late 2000s when this research took place were particularly challenging for Zimbabweans. As AN explains, referring to the impacts of hyperinflation: “…this money has been devalued so much that you cannot buy anything with it; so poverty has really limited our efforts” (AN, female, Time 2, StB). On top of hyperinflation, as AL (male, Time 2, StA) explains, the region faced several seasons of drought that severely reduced harvests: “…This year there has been drought so much that even the well-known crop producers did not harvest anything.” Poverty and drought curtailed many efforts to increase visits to provide support to PLWHA. PLWHA and their families often were in desperate need of food or money for medicine. Visiting their houses empty handed was considered both culturally inappropriate and callous, making it hard for conversation participants to implement plans of increasing social relations with PLWHA.

**KT:** I think the major challenge [in acting on our CC plans] has been poverty that made it difficult for us to meet the expectation of the patients we visited. The most important thing that we also failed to make available has been food and a decent or good diet. Unfortunately we had a very poor harvest. (female, Time 3, StB)

Poverty also reduced the capacity of community members to offer physical care to PLWHA, because carers were unable to access gloves. Since people were understandably highly reluctant to touch open sores and human waste without gloves, they were often hesitant to visit the homes of very sick people to offer assistance, further isolating PLWHA and their families.

**NA:** Sure, sometimes we go to see these HIV/AIDS sufferers and they would have messed themselves but we no longer have gloves to use we find it hard to handle that. My honest request is that if we can only get a regular supply of gloves. (male, Time 3, StC)

Moreover, poverty and hunger fuelled the risky sexual behaviour that causes HIV to spread, particularly by creating conditions in which young women engaged in transactional sex with older men:

**VE:** Our efforts to give information to young people are often hampered by poverty. Our young girls think that if they go out with older man they can get what they want and all their poverty can be a history. All these hardships associated with economic situation makes it hard for young people to change. (female, Time 3, StA)

The difficult political situation in Zimbabwe was another very salient contextual feature that frustrated participant efforts to help to PLWHA and increase discussions around HIV issues. As mentioned above, it led many NGOs to withdraw from the country, removing much needed HIV awareness programs and food supplementation initiatives. Moreover, laws requiring police permission for people to gather in groups inhibited joint activity and efforts to raise money for PLWHA:

**LA:** Some of the problems we encountered recently, while a lot of AIDS patients had openly told us their status, so we embarked on a door-to-door campaign to raise money for these patients but recently the political environment was not enabling at times being accused of trying to raise money for the opposition party. (female, Time 2, NA)

**MA:** We had a problem because visiting patients was not easy due to the tense political environment, where it was not easy for people to visit these patients as a group. (male)

**ZA**: That is true to such an extent that one day when I and a small group of church members had visited a certain patient and we were approached and told to call off the visit in case it was seen as a political gathering - rather than a small group of church members going to see a patient. (female Time 2, StA)

Community collaboration and dialogue are absolutely central to the community conversations approach-and to the idea of social change in general. Limits on community gatherings restrict peoples’ opportunities to develop or implement action plans to improve local responses to HIV.

## Conclusions

In this paper we have provided a case study of the use of community conversations to promote critical thinking and action planning in response to HIV/AIDS in Zimbabwe, using the conceptualisation of community-level ‘HIV/AIDS competence’ as a lens for analysis and action in this field [8-10]. The value of community conversations stems from their creation of social spaces for dialogue, which can enable marginalized people (in this case the impoverished rural Zimbabweans in our study) to engage in critical thinking. People must have opportunities to conceive of strategies for change. However, conversations can at most be a necessary condition for the implementation of strategies, and not a sufficient one. Our findings suggest that conversations may create social space for people to reflect on the possibility of more effective responses to HIV, but a host of other factors will intervene in shaping whether such reflection leads to concrete behaviour change, Community conversations cannot counter the effects of poverty, poor harvests and political upheaval that limit the capacity of local people to solve the problems they face. They take place within a wider social, political and economic context that plays a major role in enabling or frustrating community efforts to combat HIV. Community conversations cannot make a woman economically empowered enough to leave transactional sex work, nor can they put bus fare to the clinic in the pocket of a young man seeking an HIV test and they cannot bring ART to a rural community.

Here we seek to re-emphasise that we by no means seek to make claims about linear or causal pathways from community conversations to behaviour change. Our aim has been a more limited one: to show that conversations can indeed provide social spaces for critical discussion, and for brainstorming of strategies that could be implemented by local people using existing community resources. Furthermore, we are not seeking to make any claims about whether such strategies may or may not be implemented following the conversations. Methodologically, several features of our study would prevent us from making such claims: its modest scale, the fact that engagement in community conversations might be self-selecting of those who are the most concerned about the social issue in question, or the most willing to engage in dialogical encounters regarding sensitive topics.

Furthermore it would be premature for us to use our findings to argue that community conversations should be ‘scaled up’ for larger impact. The average public health initiative in a poor country is unlikely to have access to post-graduate trained external facilitators. The Ethiopian pioneers of this approach used trained local community members, but it remains uncertain as to whether trained local people (without the perceived status of our facilitators) would have succeeded as well in our context of interest. Irregular attendance of participants even in this relatively well-resourced and small scale pilot further suggest that assertive recommendations for scaling up the approach would be premature, although we have no doubt that poor attendance was heavily influenced by the political climate in Zimbabwe at the time of our study rather than a lack of interest or commitment by local people.

Also adding to the need for caution is a recent companion study of the processes of dialogical communication about HIV/AIDS in rural Zimbabwe, where we found that group discussions about HIV/AIDS stigma sometimes had the unintended consequence of providing an arena for people to express discriminatory attitudes in ways that were not challenged by other group members [38]. This echoes Portes and Landolt’s [39] warning that community group engagements may sometimes generate ‘anti-social capital’, and not always have positive consequences.

However, despite all these proviso’s we remain confident that our conversations were successful in the modest aims which we set them – to create spaces in which people might ‘break the silence’, think critically about obstacles to effective responses and brainstorm action plans. Such dialogue is a vital, if not a sufficient, precondition for health-enhancing behaviour change. In this respect we are confident that our study supports the need for further exploration of the potential for conversations to contribute to tackling key challenges such as making the uptake of available services seem more socially acceptable and potentially reducing the stigma of being seen in the clinic collecting ARVs. They appeared to help our participants envisage feasible concrete strategies for helping the HIV affected. They provided a forum in which to build a sense of community and common purpose, to encourage participants to conceive ways to move from HIV-related information to action, and to reduce the silence and stigma surrounding HIV, challenging them to develop constructive strategies for change.

## Endnotes

^a^The term ‘patients’ is commonly used by people in this part of Zimbabwe to refer to HIV-positive people suffering the physical decline of AIDS.

## Competing interests

The authors declare that they have no competing interests.

## Authors’ contributions

CC led the conceptualisation, design, and interpretation of the data. MN, CM and CN played a key role in data collection and management. CC and KS performed the data analysis and worked together in drafting the paper. MN, CM, MS and SG contributed to the drafting of the manuscript, or revising it critically for important intellectual content. All authors read and approved the final manuscript.

## Pre-publication history

The pre-publication history for this paper can be accessed here:

http://www.biomedcentral.com/1471-2458/13/354/prepub
